# Phase 1 study of everolimus and low-dose oral cyclophosphamide in patients with metastatic renal cell carcinoma

**DOI:** 10.1007/s00262-018-2248-3

**Published:** 2018-11-09

**Authors:** Charlotte M. Huijts, Inge M. Werter, Sinéad M. Lougheed, Ruben S. Goedegebuure, Carla M. van Herpen, Paul Hamberg, Metin Tascilar, John B. Haanen, Henk M. Verheul, Tanja D. de Gruijl, Hans J. van der Vliet, for the Dutch WIN-O Consortium

**Affiliations:** 1 Department of Medical Oncology, VU University Medical Center, Cancer Center Amsterdam, De Boelelaan 1117, 1081 HV Amsterdam, The Netherlands; 2 Department of Medical Oncology, Radboud University Medical Center, Nijmegen, The Netherlands; 3 Department of Medical Oncology, Franciscus Gasthuis, Rotterdam, The Netherlands; 4 Department of Medical Oncology, Isala Clinics, Zwolle, The Netherlands; 5 Division of Medical Oncology, Netherlands Cancer Institute, Amsterdam, The Netherlands

**Keywords:** Cyclophosphamide, Tregs, mTOR, Everolimus

## Abstract

**Abstract:**

mTOR inhibitors are frequently used in the treatment of metastatic renal cell cancer (mRCC). mTOR regulates cell growth, proliferation, angiogenesis, and survival, and additionally plays an important role in immune regulation. Since mTOR inhibitors were shown to benefit immunosuppressive regulatory T-cell (Treg) expansion, this might suppress antitumor immune responses. Metronomic cyclophosphamide (CTX) was shown to selectively deplete Tregs. This study was, therefore, designed to determine the optimal dosage and schedule of CTX when combined with everolimus to prevent this potentially detrimental Treg expansion. In this national multi-center phase I study, patients with mRCC progressive on first line anti-angiogenic therapy received 10 mg everolimus once daily and were enrolled into cohorts with different CTX dosages and schedules. Besides immune monitoring, adverse events and survival data were monitored. 40 patients, 39 evaluable, were treated with different doses and schedules of CTX. Combined with 10 mg everolimus once daily, the optimal Treg depleting dose and schedule of CTX was 50 mg CTX once daily. 23 (59%) patients experienced one or more treatment-related ≥ grade 3 toxicity, mostly fatigue, laboratory abnormalities and pneumonitis. The majority of the patients achieved stable disease, two patients a partial response. Median PFS of all cohorts was 3.5 months. In conclusion, the optimal Treg depleting dose and schedule of CTX, when combined with everolimus, is 50 mg once daily. This combination leads to acceptable adverse events in comparison with everolimus alone. Currently, the here selected combination is being evaluated in a phase II clinical trial.

**Trial registration:**

NCT01462214.

**Electronic supplementary material:**

The online version of this article (10.1007/s00262-018-2248-3) contains supplementary material, which is available to authorized users.

## Introduction

In 2017, 63,990 new cases and 14,400 deaths due to kidney cancer are estimated in the United States and thereby it belongs to the 10 most common cancers in both men and women [1]. The most common tumor arising in the kidney is renal cell carcinoma (RCC). Due to new techniques the histological classification has changed. Though clear cell, papillary and chromophobe RCC are still the most common subtypes, a total of more than 10 subtypes can now be identified [2]. The treatment of metastatic RCC (mRCC) has radically changed over the past 10 years. After years with limited treatment options, when interferon-α and interleukin-2 achieved response rates in only 10–20% of the patients, inhibitors of the vascular endothelial growth factor (VEGF)—signaling pathway and inhibitors of the mammalian target of rapamycin (mTOR), such as temsirolimus and everolimus, were introduced as first and second line treatment options respectively [3]. More recently an inhibitor of the PD-1 immune checkpoint, nivolumab [4], and cabozantinib, a multi-tyrosine kinase inhibitor of MET, AXL and VEGF [5, 6] were shown to be more effective in clinical trials compared to everolimus, thereby replacing everolimus as the standard second line therapy after VEGF targeted therapy [7]. In addition, the combination of everolimus and the multi-target tyrosine kinase inhibitor lenvatinib improved progression-free survival (PFS) in patients with mRCC compared to everolimus alone following one prior anti-angiogenic therapy [8, 9].

Everolimus was shown to be an effective inhibitor of mTOR, resulting in the inhibition of cell growth, proliferation, angiogenesis and survival of tumor cells. In addition, mTOR plays an important role in immune regulation, by balancing effector T cells and regulatory T cells (Tregs) [10–13]. Tregs are important regulators of immunological tolerance and dependent on the transcription factor FoxP3 for their immune suppressive functionality [14, 15]. mTOR inhibition was shown to result in Treg expansion [16–18] and increased levels of Tregs have been associated with poor survival in cancer patients, including mRCC [19–21]. Recently, we and others reported that everolimus leads to Treg proliferation, both in vitro and in vivo [22–24]. Metronomic administration of CTX has been reported to result in Treg depletion, with possible beneficial effects on T- and NK-cell functionality [25, 26]. Therefore, we hypothesized that addition of metronomic CTX to therapy with everolimus in patients with mRCC might counteract the detrimental Treg expansion induced by everolimus and could thereby increase the antitumor efficacy. In this phase I study we aimed to determine the optimal dose of CTX that would result in the selective depletion of Tregs when combined with a fixed dose (10 mg) of everolimus, taking into account the safety and tolerability of the combination treatment.

## Patients and methods

### Patients

Between January 2012 and August 2015, patients were enrolled in this clinical trial initiated by the department of medical oncology of the VU University Medical Center and conducted within the context of the Netherlands Working Group on Immunotherapy of Oncology (WIN-O) with participation of 13 hospitals. Main inclusion criteria for this trial were an age of 18 years or older, clear-cell mRCC and progression on treatment with a VEGF receptor tyrosine kinase inhibitor. In addition, patients had to have adequate hematologic, hepatic and renal function, measurable or evaluable disease as defined by RECIST 1.1 and a WHO performance status of 0–2. A more detailed description of in- and exclusion criteria can be reviewed in the previously published study protocol [27]. Follow-up was performed until death or at trial analysis, 2 years after inclusion of the last patient.

### Treatment

Patients were treated with different doses and schedules of low-dose oral CTX in combination with a fixed dose of everolimus once daily. CTX was either given in a week-on/week-off schedule or continuously and either once or twice daily. These doses and schedules were based on the CTX doses used by Ghiringhelli et al. [26]. Patients were enrolled in cohorts of five patients per dose level. In dose level 6, one patient stopped treatment because of several toxicities (highest grade 3 nausea) within 2 weeks of enrollment and was not evaluable. In case of severe toxicity dose reductions were allowed.

The first five patients were enrolled in an everolimus only cohort with 10 mg everolimus. Subsequently five patients were treated in cohort 1 with the combination of 10 mg everolimus and 50 mg CTX once daily, week-on/week-off. In cohort 2 patients were treated with the combination of everolimus and 50 mg CTX once daily, continuously. In cohort 3 patients received 50 mg CTX twice daily, week-on/week-off, and in cohort 4 patients received 50 mg CTX twice daily, continuously. In the last two cohorts, cohort 5 and 6, respectively, patients received 100 mg CTX twice daily, in cohort 5 in a week-on/week-off regimen and in cohort 6 continuously.

### Study objectives

The primary objectives of the study were to determine a recommended dose and schedule for metronomic cyclophosphamide which, when combined with the standard once daily oral dose of 10 mg of everolimus, resulted in optimal and selective Treg depletion in patients with mRCC and to determine the safety and tolerability of this combination. Secondary study objectives included (a) assessment of effects on various immune cell populations, (b) effects on selected angiogenesis parameters, (c) the effect of cyclophosphamide on everolimus drug levels, and (d) clinical outcome measures such as response rate, time to progression, and OS.

### Evaluation of toxicity and clinical activity

Patients were treated in cohorts of 5 patients per dose level. In case of no more than 1 dose limiting toxicity (DLT) in a cohort within the 28 days after start of the study treatment, it was allowed to proceed to the next dose level. DLTs were defined as febrile neutropenia, neutropenic infection, other grade ≥ 3 hematological toxicity, pneumonitis, nausea, vomiting, diarrhea, fatigue or any other grade ≥ 3 adverse event that, despite appropriate supportive care, failed to recover to grade ≤ 1 or baseline severity (or grade ≤ 2 at the investigator’s and sponsor’s discretion) after delaying the next cycle for up to 7 days.

Response to treatment was assessed by the use of RECIST version 1.1. Evaluable patients were defined as those patients completing at least 2 weeks of combination therapy, i.e., allowing the monitoring of immunological effects at time point 2 weeks. Furthermore, patients were evaluated for their performance status, vital signs, general laboratory parameters and immune monitoring at baseline and after 2, 4 and 8 weeks of treatment and every 4 weeks for their clinical condition and general laboratory parameters until the end of study treatment. CT scans of the chest and abdomen were made at baseline and thereafter every 8 weeks. Patients receiving any study treatment were evaluable for safety. Adverse events were graded according to the National Cancer Institute Common Toxicity Criteria (CTC) grading system version 3.0 (NCI-CTCAE v3.0).

### Immune monitoring

Peripheral blood was collected for extensive monitoring at baseline and subsequently at 2, 4, and 8 weeks after the start of the study treatment period and at the end of study treatment. For immune monitoring 60 mL of heparinized peripheral blood was collected. All material was processed on the same day the blood was drawn. In this manuscript, we present immune monitoring data of the effects of the various treatment cohorts on the induction of Treg depletion, the primary objective of this study. The effects of the various treatment cohorts on other immunological parameters will be comprehensively published separately.

PBMC were isolated from heparinized blood of patients by density-gradient centrifugation with Lymphoprep (Axis-Shield, Oslo, Norway). After isolation PBMC were stored overnight at 4 °C in RPMI 1640 (Lonza, Basel, Switzerland) supplemented with 100 IU/ml sodium penicillin (Astellas Pharma, Leiden, the Netherlands), 100 mg/ml streptomycin sulfate (Radiumfarma-Fisiofarma, Naples, Italy), 2.0 nM l-glutamine (Life Technologies, Bleiswijk, the Netherlands), 10% FBS (HyClone, Amsterdam, the Netherlands), and 0.05 mM 2-ME (Merck, Darmstadt, Germany), hereafter referred to as complete medium. The next day cells were stained for flow cytometric analysis.

PBMC were analyzed by flow cytometry using FITC-, PerCP- or allophycocyanin (APC)-labeled Abs directed against human CD3, CD4, and CD25 (all BD Biosciences, New Jersey, USA). Stainings were performed in PBS supplemented with 0.1% BSA and 0.02% sodium azide for 30 min. Intracellular staining was performed after fixation and permeabilization using a fixation/permeabilization kit according to the manufacturer’s protocol (eBioscience). For staining of FoxP3 a PE-labeled Ab against FoxP3 (clone PCH101, eBioscience) was used. Live cells were gated based on forward and side scatter and analyzed on a BD FACSCalibur (BD Biosciences) using Kaluza Analysis Software (Beckman Coulter).

### VEGF measurements

Plasma VEGF concentrations were measured in heparin plasma, frozen the day the material was received and stored at − 20 °C until analysis, using a commercially available ELISA kit (Quantikine, R&D Systems, Abingdon, UK) according to the manufacturers’ instructions. Absorbance was measured using a BioTek Synergy HT plate reader with an optical density of 450 nm.

### Statistical analysis

One-way repeated measures ANOVA was used to determine the statistical significance of differences within cohorts with Dunnett’s Multiple Comparison test as post-test. Two-way ANOVA was used to compare the mean values between cohorts. PFS was defined as time from baseline till progression or death, OS was defined as time from baseline till death. Both PFS and OS were analyzed using Kaplan–Meier curves. Differences were considered statistically significant when *p* values were ≤ 0.05, as indicated with asterisks (*p ≤ 0.05, **p < 0.01, ***p < 0.001). Statistical analyses were performed using GraphPad Prism software (version 7, 2016).

## Results

### Patient characteristics

Between January 2012 and August 2015, a total of 54 patients were screened for this study in 10 different hospitals in the Netherlands. Of these 54 patients, 10 patients did not meet the inclusion criteria while 3 patients withdrew their consent either before start or within the first 2 weeks of study treatment. In addition, 1 patient was excluded because of inadvertent administration of the wrong dose of study medication, while another patient was not evaluable due to early toxicity and subsequent interruption of study medication and withdrawal of informed consent; therefore, 39 patients were analyzed in the study. Patient characteristics are shown in Table 1 and supplementary table 1.

**Table 1:** Clinical characteristics

Characteristic	Study group (*n* = 39)
Median age—year (range)	66 (44–78)
Sex—no. (%)	
Male	25 (64)
Female	14 (36)
ECOG performance status—no. (%)	
0	14 (36)
1	20 (51)
2	4 (10)
Unknown	1 (2.6)
IMDC risk group^a^	
Favorable	4 (10)
Intermediate	24 (62)
Poor	9 (23)
Unknown	2 (5)
Median time from initial diagnosis to metastatic disease—months (range)	9 (0–134.5)
Median time from metastatic disease to start of study treatment—months (range)	17 (0.8–290)
Site of metastasis—no. (%)	
Lung	30 (77)
Lymph nodes	24 (62)
Bone	8 (21)
Kidney	7 (18)
Liver	5 (13)
Brain	1 (2,6)
Other^b^	21 (54)
Number of metastatic sites	
1	7 (18)
2	13 (33)
3	9 (23)
≥ 4	10 (26)
Previous systemic cancer therapy	
Sunitinib	33 (85)
Pazopanib	9 (23)
Sorafenib	3 (7.6)
Interferon +/_ bevacizumab	3 (7.6)
IL-2	1 (2.6)
Previous anti-angiogenic regimens—no. (%)	
1	31 (80)
≥ 1	8 (20)

^a^International mRCC Database Consortium or Heng criteria

^b^Adrenal gland, soft tissue, pleural space, muscle, peritoneum/mesenteries, pancreas, vagina, spleen, pericardial tissue

From the 39 patients, 64% were male. The median age of participating patients was 66 years, 20.5% received more than one prior line of systemic therapy, and 72% of patients were in the favorable or intermediate IMDC (International Metastatic Renal-Cell Carcinoma Database Consortium) risk group (Table 1).

Patients were discontinued from study therapy because of progression (*n* = 25, 64%), unacceptable toxicity (*n* = 12, 30%) or death (*n* = 2, 5%). Follow-up was performed until death (*n* = 36) or until time of analysis of the trial (*n* = 3).

### Treg depletion

**Fig. 1:**
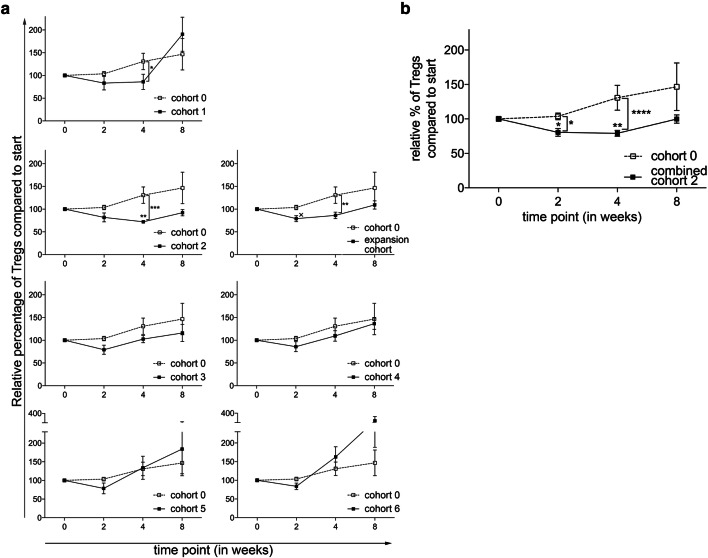
Effect of different dosages and administration schedules of CTX when combined with a fixed dose of 10 mg everolimus on the frequency of Tregs. **a** Relative percentages of Tregs within CD4^+^ T cells were determined in freshly isolated PBMC from patients treated with different dosages and schedules of CTX, combined with a fixed dose of everolimus at baseline and subsequently 2, 4, and 8 weeks after start of treatment. *p* value indicated with asterisk; *p ≤ 0.05, **p ≤ 0.01, ***p ≤ 0.001, ^x^*p* = 0.07. **b** Relative percentages of Tregs within CD4^+^ T cells are shown for cohort 2 combined with the expansion cohort. Patients were treated with 50 mg CTX once daily, combined with 10 mg everolimus once daily. Means ± SEM are shown; *p* value indicated with asterisk; *p ≤ 0.05, **p ≤ 0.01, ****p ≤ 0.0001

**Fig. 2:**
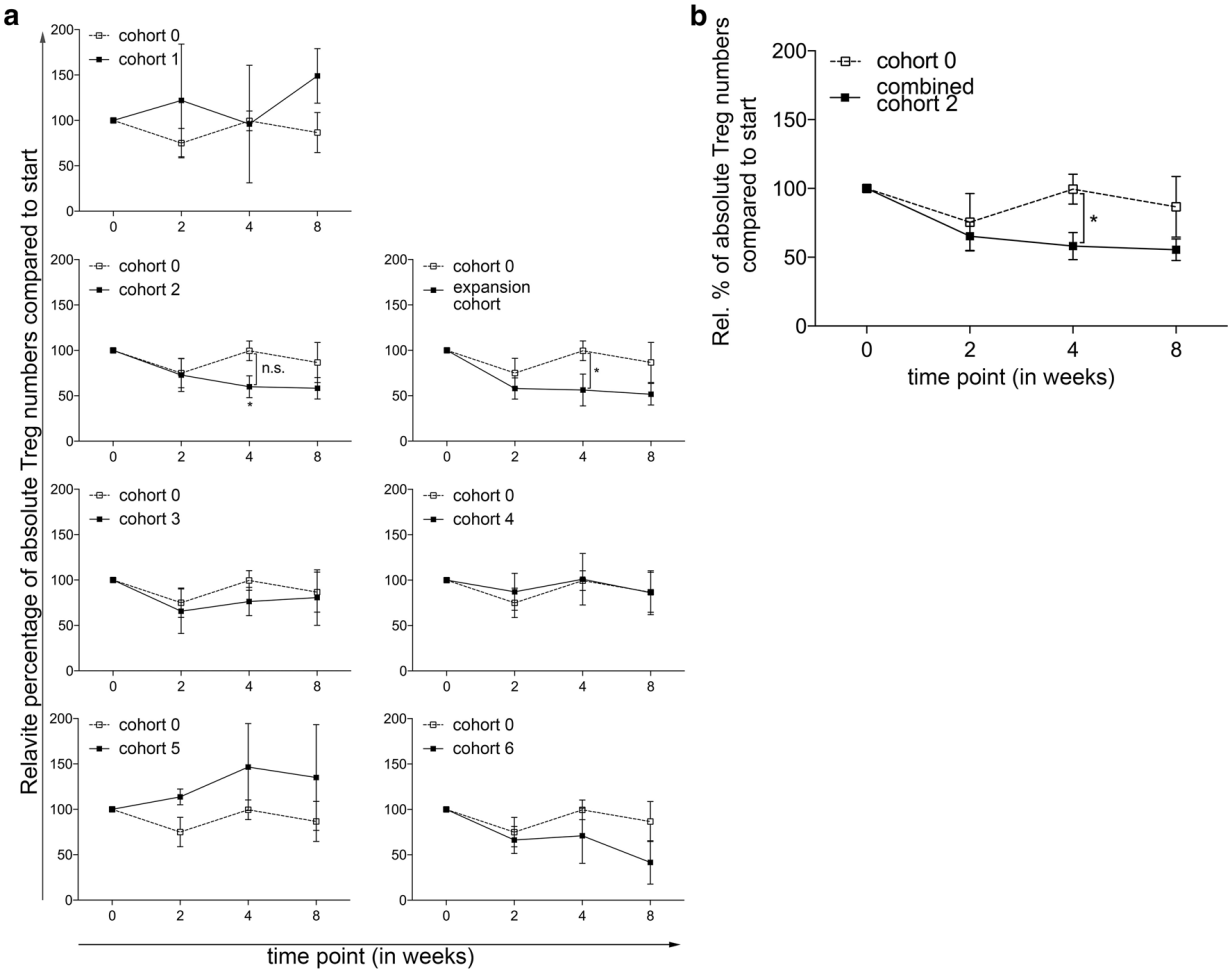
Effect of different dosages and administration schedules of CTX when combined with a fixed dose of 10 mg everolimus on absolute Treg numbers. **a** Relative percentages of absolute Treg numbers were determined in freshly isolated PBMC from patients treated with different dosages and schedules of CTX, combined with a fixed dose of everolimus at baseline and subsequently 2, 4, and 8 weeks after start of treatment. *p* value indicated with asterisk, *p ≤ 0.05. **b** Relative percentages of absolute Treg numbers are shown for cohort 2 combined with the expansion cohort. Patients were treated with 50 mg CTX once daily, combined with 10 mg everolimus once daily. Means ± SEM are shown; *p* value indicated with asterisk; *p ≤ 0.05

The main objective of this phase 1 trial was to determine the optimal dose and administration schedule of orally administered CTX, when combined with 10 mg everolimus, to obtain selective Treg depletion. As shown in Fig. 1a, a (non-significant) increase in Treg percentages within the CD4^+^ T-cell population was observed in the everolimus only cohort, cohort 0. In cohort 1, 50 mg CTX was administered in a week-on/week-off schedule. Compared to the everolimus only cohort, a significant decrease in Treg percentages at time point 4 was observed. In the next cohort, cohort 2, in which 50 mg CTX was administered in a continuous schedule, a significant decrease in Treg percentages within the cohort was observed when comparing the percentages at time point 0 to time point 4. In addition, a significant difference in Treg percentages between cohort 0 and cohort 2 was observed at time point 4, using the two-way ANOVA. Supplementary Fig. 1. shows representative flow cytometry dot plots illustrating the changes in Treg percentages. Proceeding to the following cohorts, the Treg depleting effect of CTX was progressively less pronounced. Of interest, in the last 2 cohorts, cohort 5 with administration of 100 mg CTX twice daily in a week-on/week-off schedule and cohort 6 with administration of 100 mg CTX twice daily in a continuous schedule, we even observed an increase in Treg percentages. Notably, changes in absolute Treg numbers generally followed the same patterns as observed for changes in Treg percentages. A significant decrease was observed in cohort 2 comparing Treg numbers at week 0 with week 4, while absolute Tregs numbers did not change or even increased in subsequent cohorts (Fig. 2a). Therefore, the decision was made to end the dose escalation phase of the study, and to proceed to the expansion cohort, in which an additional 5 patients were treated with the optimal Treg depleting dose observed in cohort 2. In none of the tested cohorts significant changes in CD4^+^ T cell percentages were observed. Comparing the CD4^+^ T cell percentages in the individual cohorts with cohort 0, we did find a significant difference at week 4 between cohort 0 and the expansion cohort (see Supplementary Fig. 2). Lymphocyte percentages increased within cohort 3, 4 and 5 at week 2 and decreased in cohort 6 at week 4. This resulted in significant differences between cohort 0 and cohort 5 and 6 in the first 4 weeks and only at week 4 of the study, respectively (see Supplementary Fig. 3).

The expansion cohort essentially confirmed the results previously observed in cohort 2. Again, a decrease in Treg percentages was noted between time point 0 and 4 resulting in a statistically significant difference at this time point in Treg percentages between cohort 0 and the expansion cohort. When the results of cohort 2 and the expansion cohort were combined, a highly significant decrease in the percentage of Tregs was observed, both within the combined patient cohort as well as in comparison of this cohort to cohort 0 (Fig. 1b). In absolute Treg numbers the same decrease was observed in the expansion cohort, with a significant difference at timepoint 4 between cohort 0 and the expansion cohort. When absolute number data from cohort 2 were combined with those of the expansion cohort 2E, a significant decrease in absolute Treg numbers was noted (Fig. 2b).

### Adverse events and DLT

During the entire study 314 adverse events were reported; 93 of these consisted of laboratory abnormalities (see Table 2 and supplementary table 2). The most common treatment-related toxicities (> 30%) included fatigue (*n* = 18; 46%), anorexia (*n* = 16; 41%), rash (*n* = 15; 38%), cough (*n* = 14; 36%), mucositis (*n* = 14; 36%), nausea (*n* = 12; 31%), anemia (*n* = 14; 36%), and hypercholesterolemia (*n* = 12; 31%). The mean number of adverse events of any grade was 8.2 per patient in the total group, while a mean of 5.4 adverse events per patient occurred in cohort 0 (i.e., in the cohort without CTX). When patients were treated for a longer period with the study drugs, more adverse events were reported. When adjusted, a mean of 3.2 adverse events per month was reported. After this adjustment, the two cohorts with the highest CTX dose showed slightly more adverse events compared to the lower cohorts.

**Table 2:** Treatment-related toxicity

Event	Any Grade	Number of patients (%)		
		Grade 1	Grade 2	Grade ≥ 3
Neurology				
Neuropathy	4 (10)	3 (8)	1 (3)	0
Respiratory				
Cough	14 (36)	11 (28)	3 (8)	0
Dyspnea	10 (26)	5 (13)	4 (10)	1 (3)
Pneumonitis	7 (18)	1 (3)	3 (8)	3 (8)
Gastro-intestinal				
Mucositis	14 (36)	10 (26)	4 (10)	0
Nausea	12 (31)	6 (15)	6 (15)	0
Diarrhea	11 (28)	8 (20)	1 (3)	2 (5)
Vomiting	9 (23)	4 (10)	5 (13)	0
Dysgeusia	6 (15)	4 (10)	2 (5)	0
Stomatitis	5 (13)	3 (8)	1 (3)	1 (3)
Constipation	4 (10)	1 (3)	3 (8)	0
Renal/genitourinary				
(Hemorrhagic) cystitis	7 (18)	2 (5)	4 (10)	1 (3)
Pollakisuria	4 (10)	3 (8)	1 (3)	0
Constitutional				
Fatigue	18 (46)	5 (13)	8 (20)	5 (13)
Anorexia	16 (41)	8 (20)	8 (20)	0
Fever/chills/flu	5 (13)	5 (13)	0	0
Malaise	4 (10)	2 (5)	1 (3)	1 (3)
Dermatology				
Rash	15 (38)	9 (23)	6 (15)	0
Dry skin	8 (20)	6 (15)	2 (5)	0
Pruritus	4 (10)	4 (10)	0	0
Laboratory				
Anemia	14 (36)	2 (5)	10 (26)	2 (5)
Hypercholesterolemia	12 (31)	3 (8)	7 (18)	2 (5)
Lymphocytopenia	10 (26)	0	2 (5)	8 (20)
Hyperglycemia	10 (26)	1 (3)	6 (15)	3 (8)
Thrombocytopenia	10 (26)	7 (18)	1 (3)	2 (5)
Hypertriglyceridemia	8 (20)	3 (8)	3 (8)	2 (5)
Leukocytopenia	8 (20)	2 (5)	2 (5)	4 (10)
Electrolyte disturbance^a^	7 (18)	5 (13)	0	2 (5)
Liver values increased^b^	6 (15)	2 (5)	3 (8)	1 (3)
Neutropenia	5 (13)	0	3 (8)	2 (5)
Other				
Edema (extremities/face)	4 (10)	3 (8)	0	1 (3)

Reported in 10% or more of the treated patients

^a^Hypophosphatemia, hyponatremia, hypo- and hyperkalemia, hypocalcemia

^b^Alanine aminotransferase, aspartate aminotransferase, gamma-glutamyl transferase and alkaline phosphatase

47 treatment-related ≥ grade 3 toxicities were reported in 22 patients, and these consisted mainly of laboratory abnormalities (leukocytopenia, lymphocytopenia, hyperglycemia) and fatigue. One patient suffered from grade 4 lymphopenia after 10.5 months of treatment in cohort 5 in which 10 mg everolimus was combined with 100 mg CTX twice daily in a week-on/week-off schedule. A dose reduction had already taken place because of the toxicity, which had been present at a lower grade for a longer period. The grade 4 toxicity eventually lead to the decision to stop the study medication, followed by the radiological assessment of disease progression several days later.

Two patients experienced ≥ grade 3 toxicity within the first 28 days after start of the study treatment, one grade 3 pneumonitis and one grade 3 pancytopenia in combination with hyperglycemia. The patient with the grade 3 pneumonitis was treated in cohort 1, in which 10 mg of everolimus was combined with 50 mg CTX once daily in a week-on/week-off schedule. According to the protocol everolimus was interrupted resulting in improvement of the pneumonitis. Study medication was permanently discontinued and dyspnea persisted 46 days after the initiation of treatment and the patient showed radiological signs of progressive disease 10 days later. The patient with grade 3 pancytopenia in combination with hyperglycemia was treated in cohort 5, in which 10 mg everolimus was combined with 100 mg CTX twice daily in a week-on/week-off schedule. The adverse event occurred after 12 days of study drug administration and according to the protocol the treatment was temporarily stopped. Laboratory values improved and after 9 days of interruption both study drugs were restarted at half the original dose. Although both ≥ grade 3 toxicities occurred within the first 28 days from start of combination treatment, both occurred in different cohorts. Since ≤ 1 DLTs were experienced by the 5 patients in these cohorts, further patients could be enrolled at the next dose level.

Both in cohort 2, the cohort that showed a selective Treg depletion, as well as in the similarly dose expansion cohort, three grade 3 adverse events were reported and no DLTs.

### VEGF levels

As chemotherapy was proposed to have anti-angiogenic effects in metronomic doses (reviewed in [28]), several studies showed decreased VEGF levels after treatment with metronomic CTX [29, 30]. For this study VEGF levels were measured at baseline, week 4 and (where available) week 8. The mean baseline VEGF level of all patients included in the study was 210 ± 30 pg/ml (mean ± SEM). As shown in supplementary Fig. 4, all cohorts in which patients received the combination treatment of everolimus and CTX showed lower VEGF levels during treatment as compared to cohort 0 in which patients received everolimus monotherapy. The cohorts with higher doses of CTX showed more pronounced effects; however, in neither of the cohorts, results were statistically significant.

### Clinical outcome

**Fig. 3:**
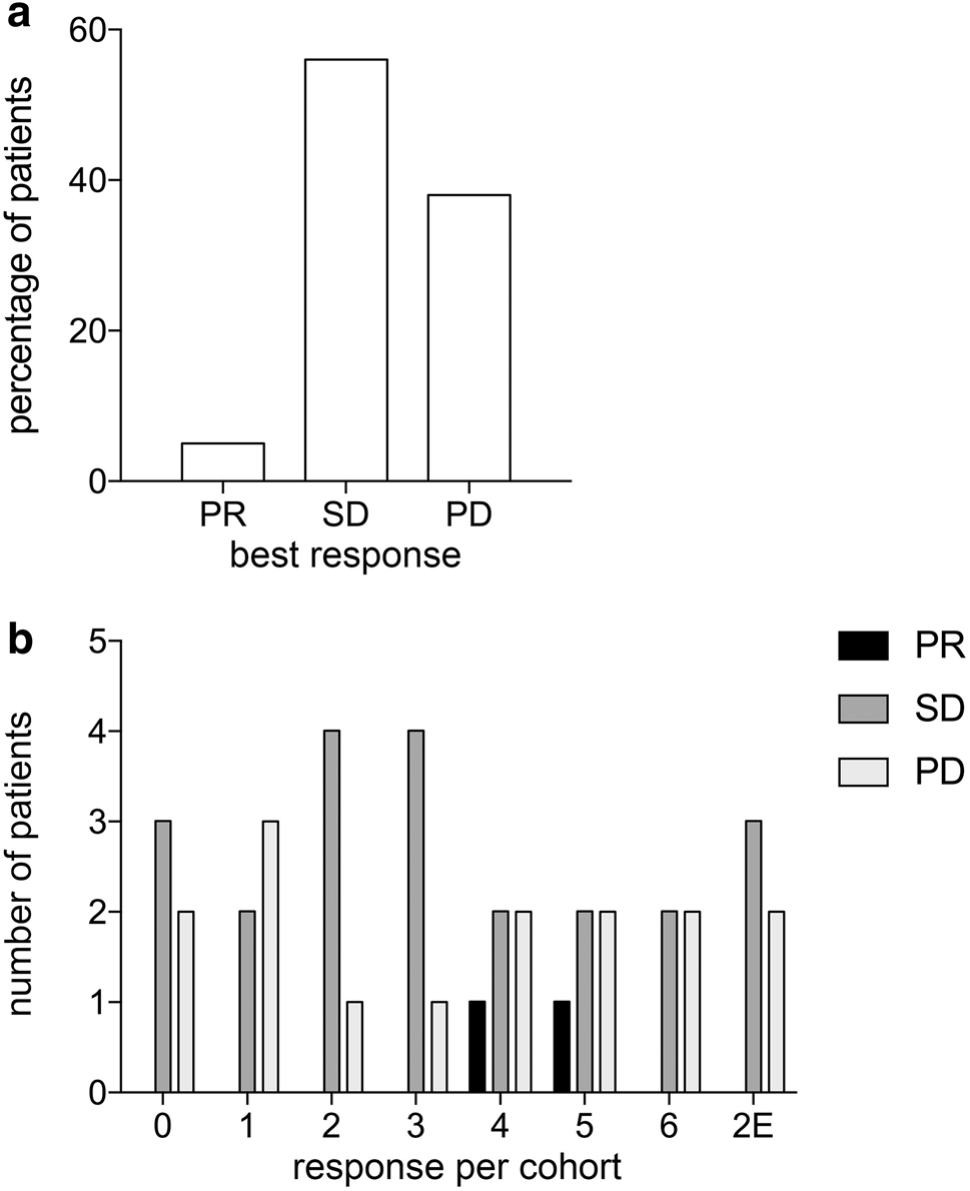
Clinical outcome. **a** Best clinical response for the total study population. **b** Best clinical response shown per cohort. Partial remission (PR) is shown in black, stable disease (SD) in grey and progressive disease (PD) in light grey

The Overall Response Rate (ORR) did not significantly differ between the investigated cohorts. The best clinical response was a partial remission (PR) in 2 patients (5%); stable disease (SD) was observed in 22 patients (56%) and progressive disease (PD) in 15 patients (39%) (Fig. 3a). The responses per cohort are shown in Fig. 3b.

**Fig. 4:**
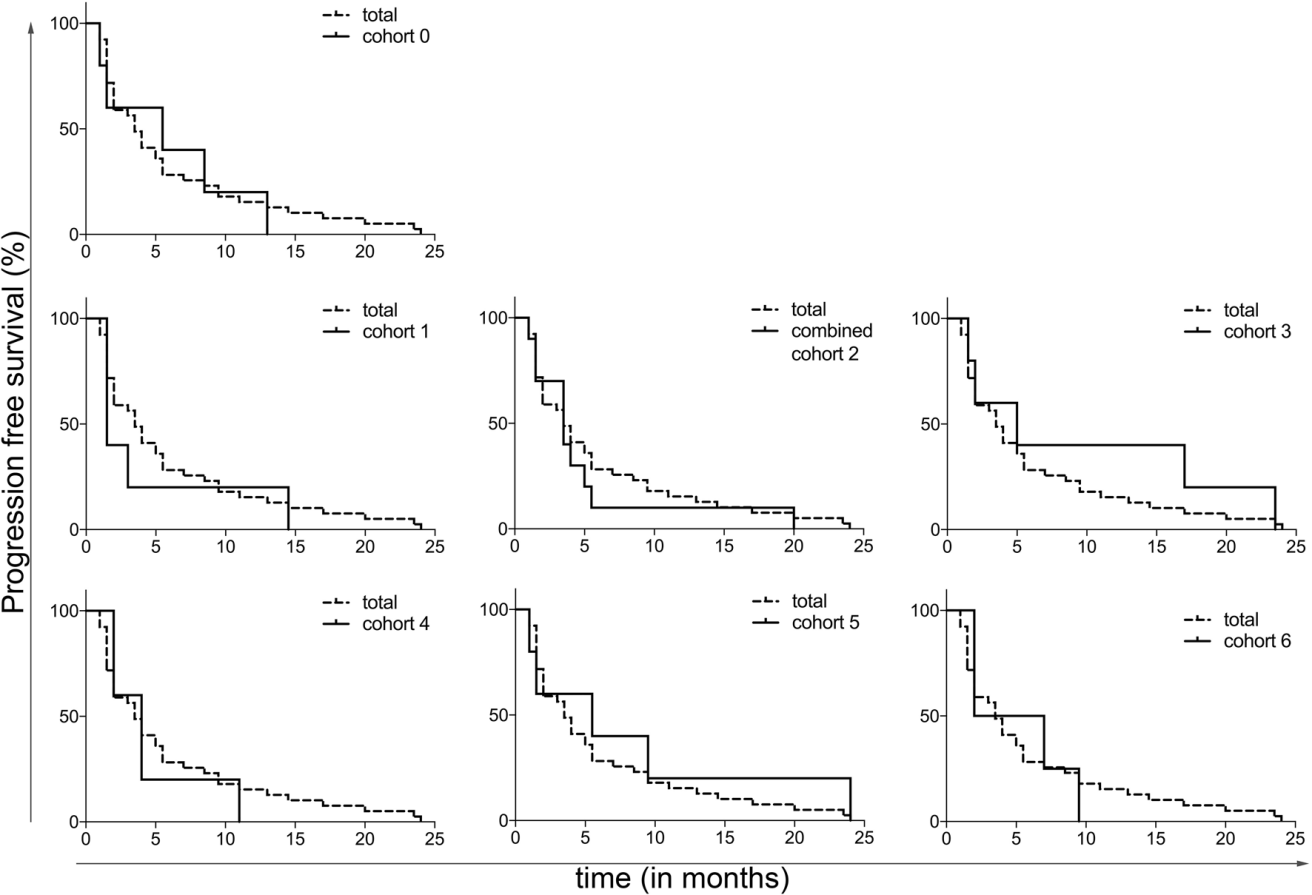
Kaplan–Meier curves for PFS per cohort, compared to the total patient group

Median PFS among all cohorts was 3.5 months (range 1–24 months). At the end of the follow-up period 1 patient did not show progression, however, this patient stopped study treatment after 8 weeks due to toxicity. After 8.5 months this patient still did not show progression, and was lost to follow-up after 25 months. No significant differences in PFS were observed between the different cohorts. In Fig. 4 the PFS is shown per cohort. There was no statistically significant correlation between Treg numbers and PFS (*R* = 0.01, *p* = 0.47; data not shown). Median OS was 11.5 months (range 1–45 months), 3 patients were still alive at the end of the follow-up period. No significant differences in OS were seen between the cohorts (see supplementary Fig. 5).

## Discussion

Since mTOR based regimens lead to Treg expansion [16–18] which can be considered an undesirable effect in the treatment of cancer, strategies that can selectively deplete Tregs might improve the antitumor effect of mTOR inhibitors by reversing the suppressive effect on the immune system. CTX was previously shown to result in selective Treg depletion [25, 26]; however, the optimal dose and schedule of metronomic CTX to induce selective Treg depletion in patients treated with mTOR inhibitors has not been determined. In the present trial, the Treg depleting effect of several dosages and schedules of metronomic CTX in combination with mTOR inhibition were investigated [27]. Our data indicate that a significant and selective Treg depletion in peripheral blood can be achieved when mRCC patients that receive the standard once daily oral dose of 10 mg everolimus are simultaneously treated with a once daily oral dose of 50 mg CTX, in a continuous scheme, whereas CD4^+^ T cell percentages remain stable. The selected dose of CTX not only resulted in a significant decrease in the frequency of Tregs but also resulted in a significant decrease in absolute Treg numbers. Surprisingly, Treg percentages were found to actually increase when higher doses of CTX were administered. Since the exact mechanism responsible for Treg depletion is unknown, similarly this resistance of Tregs to higher CTX dosages remains unclear. Several mechanisms have been proposed to be responsible for the susceptibility of Tregs to CTX. For example, Tregs were shown (1) to have low ATP levels [31] leading to reduced synthesis of glutathione and thereby decreasing the detoxification of CTX, (2) to have DNA repair defects [32] due to high levels of DNA crosslinks and (3) to have deficient expression of ABCB1 [33] making them less able to extrude CTX. On the other hand, it was shown that Tregs express aldehyde dehydrogenase (ALDH), protecting them from CTX toxicity in graft-versus-host disease [34]. However, all those mechanisms cannot completely explain the observed effects, although it might be possible that Tregs acquire increased expression of ALDH, an effect that might be accelerated when higher dosages of cyclophosphamide are administered, possibly accounting for their apparent resistance to the depleting effects of CTX at these dose levels. Whether and which of these mechanisms may underlie the observed changes in the Treg population in the patients enrolled in this trial requires further investigation.

Across all the patient cohorts that were studied, we found that the combination of everolimus and CTX resulted in toxicity comparable to that observed in the RECORD-1 trial in patients with mRCC [35]. The toxicities that were observed in our trial were all known toxicities associated with both treatment regimens. The two observed DLTs, grade 3 pneumonitis in cohort 1 and grade 3 pancytopenia in combination with hyperglycemia in cohort 5, occurred in different cohorts, and therefore, did not affect further dose escalation of CTX. Common side effects of everolimus include lymphopenia, atypical infections, non-infectious pneumonitis and elevation of serum cholesterol, glucose, and triglycerides [36]. Although these adverse events were observed in this trial, the most common side effects were fatigue, anorexia, rash, cough, mucositis, nausea, anemia, and hypercholesterolemia. Though everolimus is a known causative drug for these side effects, we cannot exclude an additional effect of CTX. All adverse events could be alleviated by adjustment of the dose of the study drug or halting the study drug, and no deaths occurred due to the study medication. All cohorts were comparable with respect to the mean number of adverse events per patient, with a mean of 8.2 per patient. When patients were treated for a longer period with the study drugs, more adverse events were reported. The two cohorts with the highest CTX dose showed slightly more adverse events compared to the lower cohorts. Interestingly, addition of CTX to everolimus resulted in lower VEGF levels compared to the cohort in which single everolimus treatment was administered. These results were not statistically significantly different, probably due to small sample sizes and missing values at timepoint 8 weeks.

As secondary endpoints, the ORR, and median PFS and OS were calculated. Since the cohorts were small, only 5 patients per cohort, the survival data were calculated for all patients combined as shown in Fig. 4 and supplementary Fig. 5, and additionally shown for all cohorts separately. While the phase 2 part of the trial will allow formal assessment of the effect of the addition of the selected once daily oral dose of 50 mg of CTX on the clinical efficacy of everolimus, the data presented here at least show no sign of inferiority compared to historical results of everolimus monotherapy in mRCC.

In conclusion, in this trial we demonstrate that administration of 50 mg CTX once daily in a continuous schedule leads to depletion of Tregs when combined with 10 mg everolimus once daily, with toxicity comparable to that reported in the RECORD-1 trial. The treatment combination is currently under investigation in a phase 2 trial, to determine if the observed Treg depletion also results in an enhancement of the survival of patients with mRCC when compared to everolimus alone. Recently everolimus was replaced by both nivolumab and cabozantinib as the standard second line treatment for patients with mRCC [7]. In case the phase 2 part of the trial shows beneficial effects on survival, combination therapy of CTX and everolimus could still be implemented in a later treatment line. However, when everolimus is combined with lenvatinib the additional effect of CTX might be limited as, e.g. the tyrosine kinase inhibitor sunitinib, that like lenvatinib inhibits VEGF and other receptors [37, 38], was previously shown to decrease Treg frequencies [39, 40]. Besides, a sequential treatment schedule of everolimus and cyclophosphamide could be proposed, which might result in reduced Treg levels with less toxicity. Since CTX is a well-known and broadly used drug, there is much experience in the application of this drug. In addition, it is cheap, which is an advantage especially when compared to the cost of recently developed novel therapeutics. Furthermore, since everolimus is registered for the treatment of pancreatic neuroendocrine tumors, these patients might also benefit from the same treatment combination [41].

### Electronic supplementary material

Below is the link to the electronic supplementary material.

Supplementary material 1 (PDF 2847 KB)

## Abbreviations

ALDH: Aldehyde dehydrogenase
APC: Allophycocyanin
CCMO: Central Committee on Research Involving Human Subjects
CTC: Common Toxicity Criteria
CTX: Cyclophosphamide
DLT: Dose limiting toxicity
ICH: International Conference on Harmonization
IMDC: International Metastatic Renal-Cell Carcinoma Database Consortium
mRCC: Metastatic renal cell cancer
mTOR: Mammalian target of rapamycin
NCI-CTCAE v3.0: National Cancer Institute Common Toxicity Criteria (CTC) grading system version 3.0
ORR: Overall response rate
PD: Progressive disease
PFS: Progression-free survival
PR: Partial response
RCC: Renal cell carcinoma
SD: Stable disease
Treg: Regulatory T cell
VEGF: Vascular endothelial growth factor
WIN-O: Dutch Working Group on Immunotherapy of Oncology
