# Supplementary material for: Phase 1 study of everolimus and low-dose oral cyclophosphamide in patients with metastatic renal cell carcinoma
Source: Cancer Immunol Immunother. 2018 Nov 9;68(2):319–29. doi: 10.1007/s00262-018-2248-3 (PMC6394488; doi:10.1007/s00262-018-2248-3)

Supplementary table 1. Blood measurements

Measurements	Value (range)			
	Baseline	t=2	t=4	t=8
Hemoglobin (mmol/L)	7,5 (5,2-10,3)	7,2 (5,3-9,2)	6,8 (4,8-9,2)	6,5 (5,2-9,2)
WBC (x 10^9/L)	6,7 (2,9-11,1)	4,7 (2,2-7,5)	5,4 (1,8-12,3)	5,2 (1,4-13,4)
Neutrophils (x 10^9/L)	4,4 (1,7-9,6)	3 (1,3-6,2)	3,6 (1,1-8,5)	3,7 (0,9-12,1)
Eosinophils (x 10^9/L)	0,1 (0-0,5)	0,2 (0,05-0,4)	0,16 (0,03-0,62)	0,2 (0-0,64)
Basophils (x 10^9/L)	0,05 (0-0,1)	0,05 (0-0,1)	0,06 (0-0,18)	0,05 (0-0,1)
Lymphocytes (x 10^9/L)	1,5 (0,5-4,2)	1,1 (0,4-3,1)	1 (0,3-2,23)	0,8 (0,09-2,1)
Monocytes (x 10^9/L)	0,61 (0,2-1,15)	0,4 (0,1-0,8)	0,5 (0,2-1,47)	0,5 (0,02-1,15)
Platelets (x 10^9/L)	286 (121-585)	197 (47-528)	273 (85-691)	256 (76-684)
Corrected Calcium (mmol/L)	2,5 (2,2-3)	2,4 (2,1-2,7)	2,5 (2,2-3,3)	2,4 (2,1-3,0)
LDH (U/L)	250 (80-2133)	240 (80-1734)	341 (107-2614)	263 (113-454)

Supplementary table 2. Treatment-related toxicity per cohort[1]

Event	All cohorts	Cohort 0	Cohort 1	Cohort 2	Cohort 3	Cohort 4	Cohort 5	Cohort 6	Cohort 2E
	Number of patients								
Neuropathy									
**Any Grade**	**4 (10%)**	**2**	**1**			**1**			
Grade 1	3 (8%)	1	1			1			
Grade 2	1 (3%)	1							
Grade ≥3	0								
Cough									
**Any Grade**	**14 (36%)**		**3**	**4**	**1**		**3**	**2**	**1**
Grade 1	11 (28%)		2	3	1		2	2	1
Grade 2	3 (8%)		1	1			1		
Grade ≥3	0								
Dyspnea									
**Any Grade**	**10 (26%)**	**1**	**3**	**2**			**1**	**2**	**1**
Grade 1	5 (13%)	1	1	2					1
Grade 2	4 (10%)		1				1	2	
Grade ≥3	1 (3%)		1						
Pneumonitis									
**Any Grade**	**7 (18%)**		**3**	**2**	**1**		**1**		
Grade 1	1 (3%)			1					
Grade 2	3 (8%)		1	1	1				
Grade ≥3	3 (8%)		2				1		
Mucositis									
**Any Grade**	**14 (36%)**	**1**	**1**	**1**	**3**	**2**	**1**	**2**	**3**
Grade 1	10 (26%)	1		1		2	1	2	3
Grade 2	4 (10%)		1		3				
Grade ≥3	0								
Nausea									
**Any Grade**	**12 (31%)**	**1**	**1**			**3**	**2**	**2**	**3**
Grade 1	6 (15%)		1			2		1	2
Grade 2	6 (15%)	1				1	2	1	1
Grade ≥3	0								
Diarrhea									
**Any Grade**	**11 (28%)**	**2**	**2**		**1**	**1**	**1**	**1**	**3**
Grade 1	8 (20%)	1	2		1			1	3
Grade 2	1 (3%)	1							
Grade ≥3	2 (5%)					1	1		
Vomiting									
**Any Grade**	**9 (23%)**	**1**	**1**			**2**	**2**	**2**	**1**
Grade 1	4 (10%)		1			1	1	1	
Grade 2	5 (13%)	1				1	1	1	1
Grade ≥3	0								
Dysgeusia									
**Any Grade**	**6 (15%)**	**1**		**2**			**1**	**1**	**1**
Grade 1	4 (10%)	1		1			1		1
Grade 2	2 (5%)			1				1	
Grade ≥3	0								
Stomatitis									
**Any Grade**	**5 (13%)**		**1**	**2**	**1**	**1**			
Grade 1	3 (8%)			1	1	1			
Grade 2	1 (3%)		1						
Grade ≥3	1 (3%)			1					
Constipation									
**Any Grade**	**4 (10%)**							**1**	**3**
Grade 1	1 (3%)								1
Grade 2	3 (8%)							1	2
Grade ≥3	0								
(Hemorrhagic) cystitis									
**Any Grade**	**7 (18%)**			**1**		**1**	**2**	**2**	**1**
Grade 1	2 (5%)						1		1
Grade 2	4 (10%)			1		1	1	1	
Grade ≥3	1 (3%)							1	
Pollakiuria									
**Any Grade**	**4 (10%)**					**1**		**2**	**1**
Grade 1	3 (8%)							2	1
Grade 2	1 (3%)					1			
Grade ≥3	0								
Fatigue									
**Any Grade**	**18 (46%)**	**2**	**2**	**4**	**1**		**3**	**2**	**4**
Grade 1	5 (13%)		2	2			1		
Grade 2	8 (20%)	1		2	1		1	1	2
Grade ≥3	5 (13%)	1					1	1	2
Anorexia									
**Any Grade**	**16 (41%)**	**3**	**1**	**2**		**4**	**1**	**2**	**3**
Grade 1	8 (20%)	2	1	1		2	1		1
Grade 2	8 (20%)	1		1		2		2	2
Grade ≥3	0								
Fever/chills/flu									
**Any Grade**	**5 (13%)**			**2**	**2**	**1**			
Grade 1	5 (13%)			2	2	1			
Grade 2	0								
Grade ≥3	0								
Malaise									
**Any Grade**	**4 (10%)**	**1**		**1**					**2**
Grade 1	2 (5%)	1							1
Grade 2	1 (3%)			1					
Grade ≥3	1 (3%)								1
Rash									
**Any Grade**	**15 (38%)**	**1**	**1**	**2**	**4**	**2**	**2**	**1**	**2**
Grade 1	9 (23%)			1	2	2	1	1	2
Grade 2	6 (15%)	1	1	1	2		1		
Grade ≥3	0								

Event	All cohorts	Cohort 0	Cohort 1	Cohort 2	Cohort 3	Cohort 4	Cohort 5	Cohort 6	Cohort 2E
				Number of patients					
Dry skin									
**Any Grade**	**8 (20%)**			**1**	**3**	**2**	**1**		**1**
Grade 1	6 (15%)			1	1	2	1		1
Grade 2	2 (5%)				2				
Grade ≥3	0								
Pruritus									
**Any Grade**	**4 (10%)**				**2**	**2**			
Grade 1	4 (10%)				2	2			
Grade 2	0								
Grade ≥3	0								
Anemia									
**Any Grade**	**14 (36%)**	**1**	**1**	**3**	**1**	**1**	**3**	**2**	**2**
Grade 1	2 (5%)	1	1						
Grade 2	10 (26%)			2	1	1	2	2	2
Grade ≥3	2 (5%)			1			1		
Hypercholesterolemia									
**Any Grade**	**12 (31%)**	**1**	**1**	**2**	**4**	**1**		**1**	**2**
Grade 1	3 (8%)				1				2
Grade 2	7 (18%)	1		2	2	1		1	
Grade ≥3	2 (5%)		1		1				
Lymphocytopenia									
**Any Grade**	**10 (26%)**		**1**		**1**	**2**	**3**	**3**	
Grade 1	0								
Grade 2	2 (5%)						1	1	
Grade ≥3	8 (20%)		1		1	2	2	2	
Hyperglycemia									
**Any Grade**	**10 (26%)**	**1**	**2**	**2**		**1**	**2**	**1**	**1**
Grade 1	1 (3%)								1
Grade 2	6 (15%)		2	2		1		1	
Grade ≥3	3 (8%)	1					2		
Thrombocytopenia									
**Any Grade**	**10 (26%)**		**1**	**1**	**3**	**2**	**2**		**1**
Grade 1	7 (18%)		1	1	2	1	1		1
Grade 2	1 (3%)				1				
Grade ≥3	2 (5%)					1	1		
Hypertriglyceridemia									
**Any Grade**	**8 (20%)**	**1**	**2**	**1**	**2**		**1**		**1**
Grade 1	3 (8%)	1	1		1				
Grade 2	3 (8%)			1			1		1
Grade ≥3	2 (5%)		1		1				
Leukocytopenia									
**Any Grade**	**8 (20%)**			**1**	**1**	**1**	**1**	**1**	**3**
Grade 1	2 (5%)								2
Grade 2	2 (5%)			1					1
Grade ≥3	4 (10%)				1	1	1	1	
*Electrolyte disturbance**									
**Any Grade**	**7 (18%)**	**2**				**1**		**1**	**3**
Grade 1	5 (13%)	2							3
Grade 2	0								
Grade ≥3	2 (5%)					1		1	
*Liver values increased***									
**Any Grade**	**6 (15%)**		**1**		**1**	**1**	**1**	**2**	
Grade 1	2 (5%)							2	
Grade 2	3 (8%)		1			1	1		
Grade ≥3	1 (3%)				1				
Neutropenia									
**Any Grade**	**5 (13%)**				**1**	**1**	**1**	**1**	**1**
Grade 1	0								
Grade 2	3 (8%)						1	1	1
Grade ≥3	2 (5%)				1	1			
Edema (extremities/face)									
**Any Grade**	**4 (10%)**	**1**			**1**	**1**		**1**	
Grade 1	3 (8%)	1			1			1	
Grade 2	0								
Grade ≥3	1 (3%)					1			

[1] Reported in 10% or more of the treated patients

* *Hypophosphatemia, hyponatremia, hypo- and hyperkalemia, hypocalcemia*

** *Alanine aminotransferase, aspartate aminotransferase, gamma-glutamyl transferase and alkaline phosphatase*

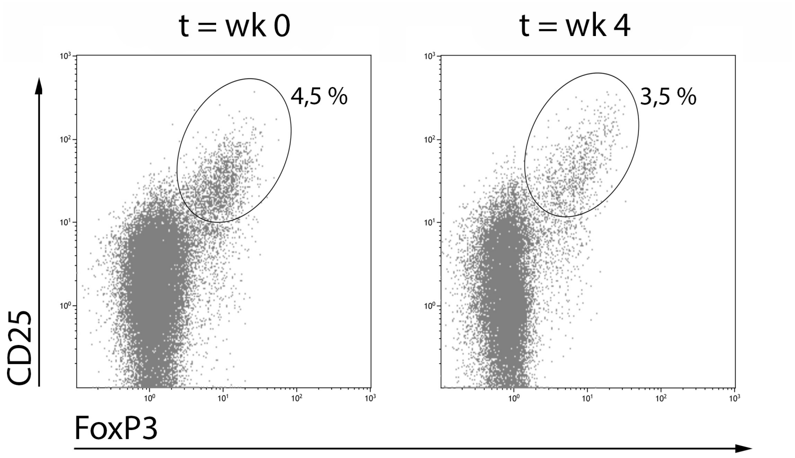

Supplementary fig. 1. Representative flow cytometry dot plots illustrating the changes in Tregs, defined as $CD3^{+}CD4^{+}CD25^{hi}FoxP3^{+}$

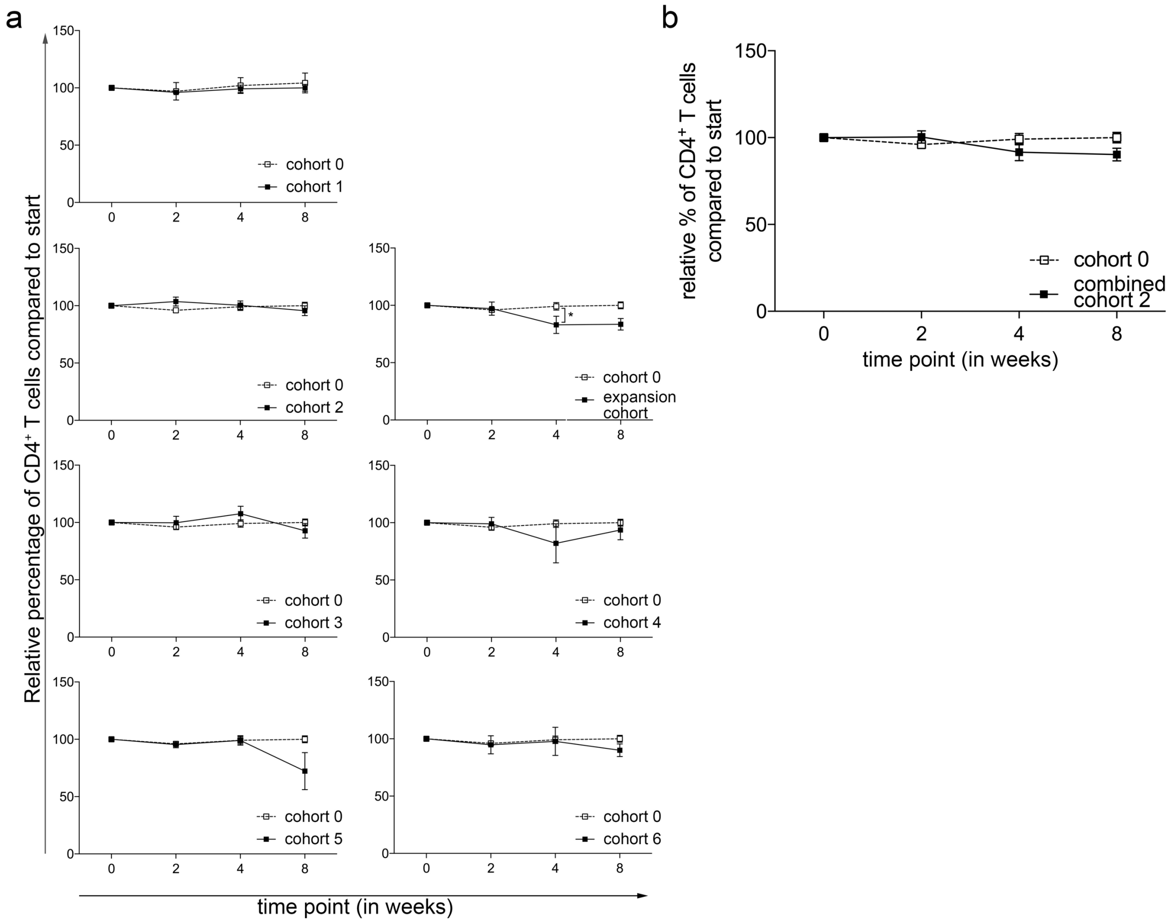

Supplementary fig. 2. Effect of different dosages and administration schedules of CTX when combined with a fixed dose of 10 mg everolimus on the frequency of $CD4^+$ T cells. a, Relative percentages of $CD4^+$ T cells within $CD3^+$ T cells were determined in freshly isolated PBMC from patients treated with different dosages and schedules of CTX, combined with a fixed dose of everolimus at baseline and subsequently 2, 4, and 8 weeks after start of treatment. b, Relative percentages of $CD4^+$ T cells within $CD3^+$ T cells are shown for cohort 2 combined with the expansion cohort. Patients were treated with 50 mg CTX once daily, combined with 10 mg everolimus once daily. Means ± SEM are shown; p-value indicated with asterisk; * $p \leq 0.05$.

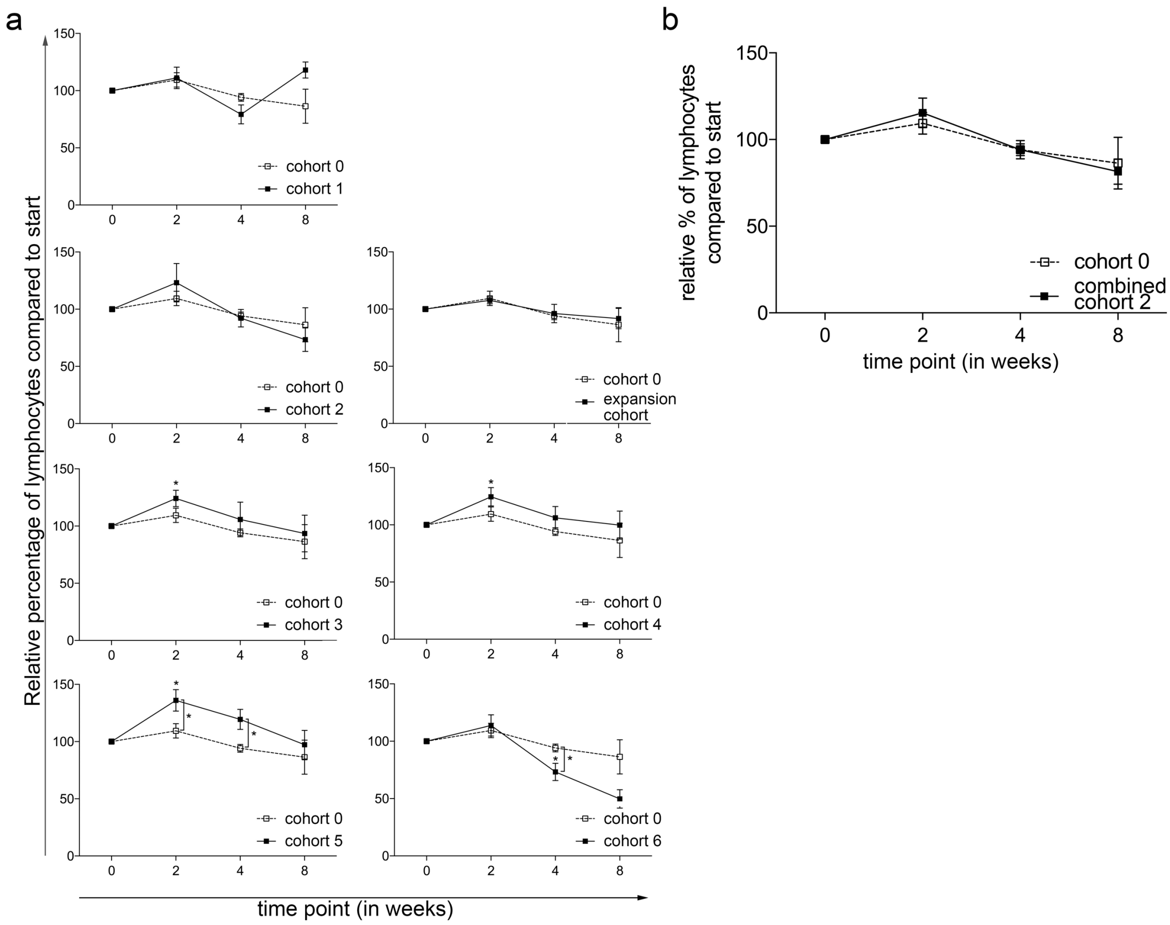

Supplementary fig. 3. Effect of different dosages and administration schedules of CTX when combined with a fixed dose of 10 mg everolimus on the frequency of lymphocytes. a, Relative percentages of lymphocytes were determined in freshly isolated PBMC from patients treated with different dosages and schedules of CTX, combined with a fixed dose of everolimus at baseline and subsequently 2, 4, and 8 weeks after start of treatment. b, Relative percentages of lymphocytes (within PBMC) are shown for cohort 2 combined with the expansion cohort. Patients were treated with 50 mg CTX once daily, combined with 10 mg everolimus once daily. Means ± SEM are shown; p-value indicated with asterisk; * $p \leq 0.05$.

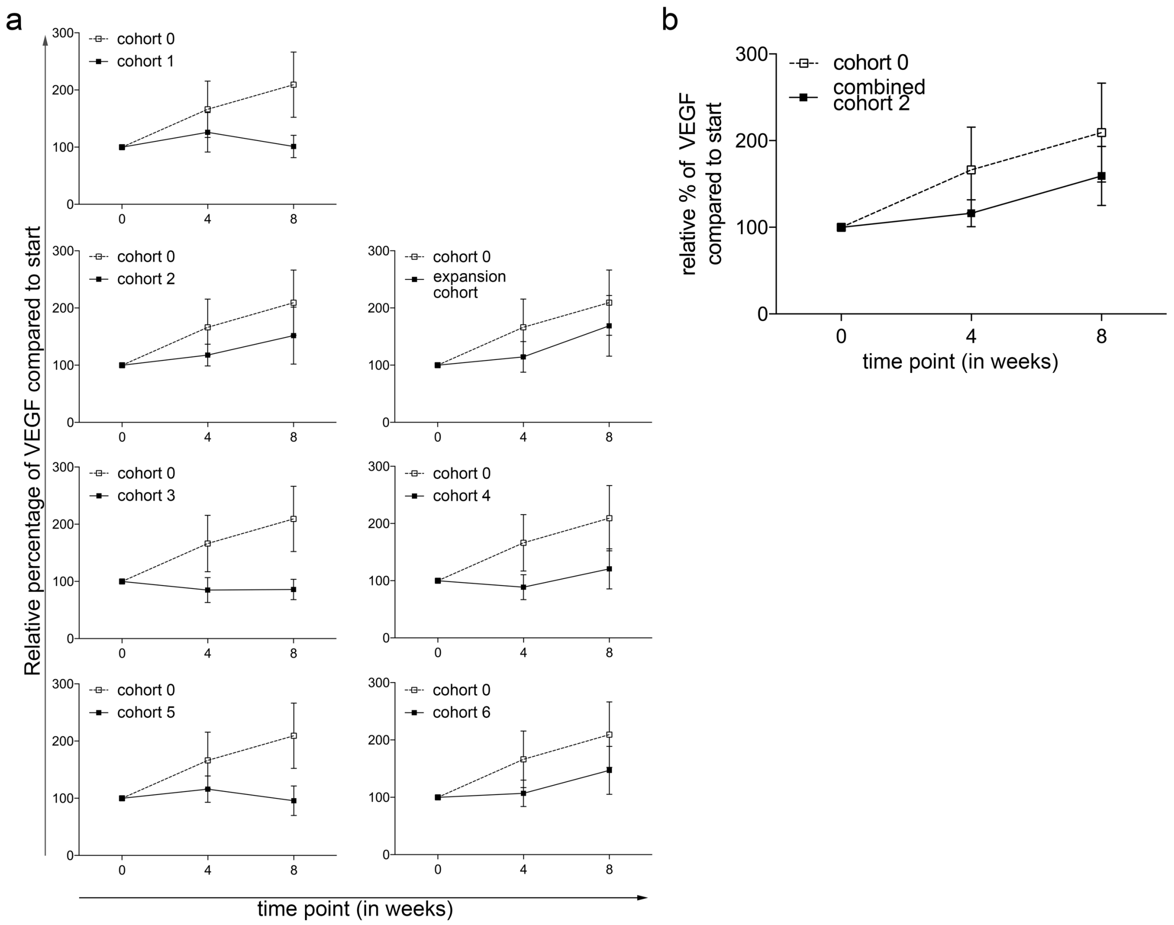

Supplementary fig. 4. Effect of different dosages and administration schedules of CTX when combined with a fixed dose of 10 mg everolimus on VEGF levels in plasma. a, VEGF levels were determined in heparin plasma at baseline and subsequently 4, and 8 weeks after start of treatment. Here, relative percentages are shown. Baseline VEGF levels (mean ± SD) per cohort: cohort 0 – 286 ± 193 pg/mL, cohort 1 – 255 ± 128 pg/mL, cohort 2 – 139 ± 104 pg/mL, cohort 3 – 122 ± 17 pg/mL, cohort 4 – 217 ± 65 pg/mL, cohort 5 – 362 ± 113 pg/mL, cohort 6 – 174 ± 27 pg/mL b, Relative percentages of VEGF plasma levels are shown for cohort 2 combined with the expansion cohort. Patients were treated with 50 mg CTX once daily, combined with 10 mg everolimus once daily. Mean VEGF level was 133 ± 25 pg/mL.

Means ± SEM are shown. No significant changes were observed.

Supplementary fig. 5. Kaplan-Meier curves for OS per cohort, compared to the total patient group.

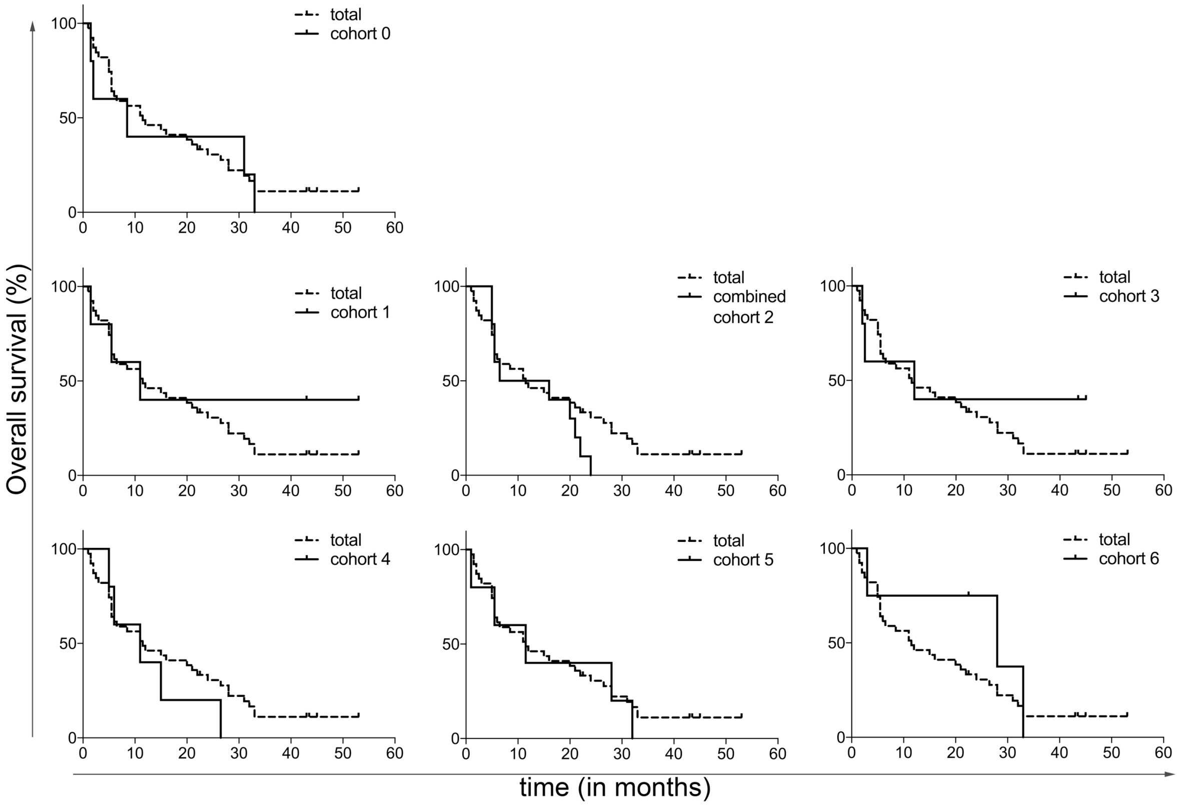

Supplement: Supplementary file 1 — Supplementary material 1 (PDF 2847 KB) [file 262_2018_2248_MOESM1_ESM.pdf]
